# Hospital Sunday and Some Critics

**Published:** 1909-06-19

**Authors:** 


### HOSPITAL ADMINISTRATION.

### CONSTRUCTION AND ECONOMICS.

### HOSPITAL SUNDAY AND SOME CRITICS.

THE PRESSING NECESSITY FOR INDIVIDUAL SELF-RELIANCE.

We desire to congratulate the Secretary, Sir Edmund Hay Currie, on the improved system of advertising this Fund which has marked the organisation in connection with Hospital Sunday, 1909. The metropolitan press, too, has taken a more active interest in the matter this year than usual, though there is still much to desire in this respect. After all, the editors of the London newspapers are Londoners; it is their especial privilege to act as leaders of public opinion; and it is not too much to expect that, although the London editors have to survey the whole Empire and world, and that in mid-June they are overdone with "copy" of all kinds, still they might and ought to devote space in a prominent position, once a year during the six days immediately preceding Hospital Sunday, in order to focus public sentiment and opinion in favour of the voluntary hospital system. Seeing that the Conference of the representatives of the press throughout the Empire has been so marked a success, would it not be desirable to have a conference of the metropolitan editors in regard to this question, and so to come to some definite and purposeful arrangement whereby the needs and claims of the whole of the hospitals of the metropolis may be adequately dealt with in the columns of the London press each year in connection with the Hospital Sunday Fund collections? The present claims to be a progressive age, and we throw out this suggestion in the hope that it may find general acceptance and lead to practical results.

We are glad to see that the clergy and ministers of religion are adopting in greater numbers the "golden pheasant" circular, in the form of a special letter addressed to individual members of their congregations, asking them, whether they are in town or not, to remember Hospital Sunday and send some contribution to the collection in accordance with their means. One such letter is before us as we write, and in it the vicar of a West End parish points out that through the agency of this Fund an incalculable amount of suffering is saved the community, and that thousands are now working and maintaining themselves and their families who, but for the help they have received through the instrumentality of the Hospital Sunday Fund, might have become invalided and entirely dependent upon charity or the Poor Law. In this connection it may be well to re-state the marvellous fact that, owing to the improved conditions in treatment and practice in the metropolitan hospitals at the present time, at least one million days every year are added to the earning power of the working classes through the diminished period of residence in the hospitals now-a-days compared with what it was a quarter of a century ago.

Reverting to the press and the writers on hospital subjects, we may perhaps urge that when a large sum is needed for a public purpose, criticism is not called for, seeing that the aim, if the writer is sincere in wishing to help, is at the moment to secure cash for the support of the hospitals. In the present year some of the criticism, which shows a socialistic bent, is neither convincing nor logical. One writer in the *Daily News*, for example, argues that, as an enormous amount of disease is preventable, the whole of the hospital system ought to be placed under the direction of the medical officers of health throughout the country and paid for by the municipality or County Council. If this were done, it is contended, the responsibility of treating the patients being placed upon the public health authorities, the tendency would be to make these gentlemen infinitely more keen than they are at present to eradicate bad housing and overcrowding, and to disperse the existing ignorance and neglect which promote a bad industrial condition.

It is not quite the time to ask the public to consent to have a million or two pounds added to the sum which they have already to provide in London each year through the rates. Indeed, the proposal displays a lack of apprehension of the facts, and of the requirements of the medical service in regard to the treatment of disease in hospitals, which is a little remarkable in the present day. Again the writer, in support of his argument, dwells upon the recent action of the Council of Newcastle-upon-Tyne in dismissing its staff of women sanitary inspectors and health visitors, who have been most efficient servants, on the ground of the cost entailed. The saving in expense by the action of the Newcastle Council cannot exceed a few hundreds a year, but, whilst it exhibits the unwisdom of spasmodic attempts at economy in municipal affairs, it certainly gives no encouragement to the claim of the *Daily News* that the whole cost of maintaining the hospitals should be provided by the municipality.

Some people have an idea that everything should be provided for them out of the taxes and rates. This present-day spirit is one of unadulterated degeneracy. England has become the great nation she undoubtedly is through the thrift and self-reliance of her people. To-day thrift and self-reliance are unpopular. Unless responsible statesmen intervene, the time is not far distant when an attempt may be made, not only to put the whole of the hospitals on the rates, but to provide everybody having less, say, than £100 a year with food, clothing, education, housing, and holidays. Whenever the shoe of necessity pinches the foot of a certain kind of person, whose numbers are steadily growing, his first impulse, instead of taking the shoe off himself and finding a remedy, is to start a grievance, because the State has not foreseen his discomfort and provided for him against it.

We have thought it well in these days of discouragement for voluntary hospital managers, whose difficulties have been immensely increased by the present Budget proposals, to call attention to the trend of individual opinion in matters of State interference, that they may use their influence in the direction of the inculcation of thrift and self-reliance on the part of the individual citizen. Be this as it may, there now exists a very general feeling of alarm and dissatisfaction in the minds of the most thoughtful and responsible residents in these islands which is quite apart from politics. The spirit of self-reliant individualism has been the very bulwark of our national prosperity in the past. When nurses, who are not by any means the best paid of women workers, have shown that they are capable in eighteen years of putting by, out of their relatively small earnings, for old-age and sickness provision and savings, a sum of something like two millions sterling; a national policy of old-age pensions, not resting on a thrift basis, certainly seems a step in the wrong direction.

